# Supplementary material for: Inhibitory Effects of Loganin on Adipogenesis In Vitro and In Vivo
Source: Int J Mol Sci. 2023 Mar 1;24(5):4752. doi: 10.3390/ijms24054752 (PMC10003152; doi:10.3390/ijms24054752)
Supplement: Supplementary file 1 [file ijms-24-04752-s001.zip › ijms-2159579-supplementary.pdf]

Inhibitory Effects of Loganin on Adipogenesis In Vitro and In Vivo

Hyoju Jeon [1,2,†], Chang-Gun Lee [1,2,†,‡], Hyesoo Jeong [3], Seong-Hoon Yun [3], Jeonghyun Kim [1,2], Laxmi Prasad Uprety [1,2], Kang-Il Oh [1,2], Shivani Singh [1,2], Jisu Yoo [1,2], Eunkuk Park [1,2,*] and Seon-Yong Jeong [1,2,3,*]

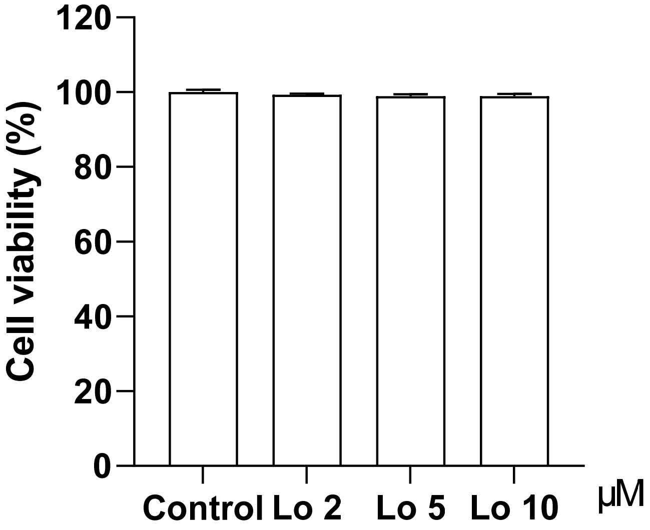

Supplementary Figure S1. Cellular viability test for loganin on 3T3-L1 cell.
